# Carbonic Anhydrase IX in Renal Cell Carcinoma, Implications for Disease Management

**DOI:** 10.3390/ijms21197146

**Published:** 2020-09-28

**Authors:** Jean Courcier, Alexandre de la Taille, Maya Nourieh, Ingrid Leguerney, Nathalie Lassau, Alexandre Ingels

**Affiliations:** 1 Biomaps, UMR1281, INSERM, Centre National de la Recherche Scientifique (CNRS), Commissariat à l’Energie Atomique (CEA), Université Paris Saclay, 94800 Villejuif, France; jean.courcier@gmail.com (J.C.); ingrid.leguerney@gustaveroussy.fr (I.L.); nathalie.lassau@gustaveroussy.fr (N.L.); 2 Department of Urology, Henri Mondor Hospital, Université Paris Est Créteil (UPEC), 94000 Créteil, France; alexandre.de-la-taille@aphp.fr; 3 Department of Pathology, Henri Mondor Hospital, UPEC, 94000 Créteil, France; maya.nourieh@aphp.fr; 4 Department of Imaging, Institute Gustave Roussy, 94800 Villejuif, France

**Keywords:** carbonic anhydrase IX, renal cell carcinoma, clear cell, immunohistochemistry, molecular imaging, targeted therapy

## Abstract

Carbonic Anhydrase IX (CAIX) is a well-described enzyme in renal cell carcinoma, with its expression being regulated by the hypoxia-inducible factor 1 alpha, it is known for interfering with hypoxia processes. Renal carcinoma encompasses a broad spectrum of histological entities and is also described as a heterogeneous malignant tumor. Recently, various combinations of checkpoint inhibitors and targeted therapies have been validated to manage this disease. Reliable markers to confirm the diagnosis, estimate the prognosis, predict or monitor the treatment response are required. Molecular imaging developments allow a comprehensive analysis of the tumor, overcoming the spatial heterogeneity issue. CAIX, being highly expressed at the tumor cell surfaces of clear cell renal carcinoma, also represents a potential treatment target. In this manuscript we reviewed the current knowledge from the literature on the pathophysiological interactions between renal cell carcinoma and CAIX, the role of CAIX as a marker for diagnosis, prognosis, treatment monitoring and molecular imaging, and the potential target for therapeutic strategies.

## 1. Introduction

Carbonic anhydrase IX (CAIX) is a member of the 14 carbonic anhydrase enzymes isoforms found in humans. It is over-expressed in many types of cancer. Renal cell carcinoma (RCC) represents around 3% of all cancers [1]. Its incidence has increased at a pace of around 2%/year for the last two decades, mostly due to the use of abdominal imaging in routine clinical practices leading to incidental diagnosis of renal tumors. There were some 99,200 new RCC cases and 39,100 kidney cancer-related deaths in 2018 within the European Union [2]. RCC encompasses many different forms of histopathological entities described in the 2016 World Health Organization classification [3]. However, there are three main RCC types: clear cell (ccRCC), papillary (Prcc-type 1 and 2) and chromophobe (chRCC).

CAIX expression was first reported in RCC in 1986, it was then named G250. Since then, many explorations have been performed to define the role of CAIX in RCC and its potential as a diagnostic, prognostic and therapeutic target to manage this disease. The aim of this review was to collect and synthetize the current knowledge on this marker in RCC pathophysiology, diagnostic and treatment.

## 2. Evidence Acquisition

We performed a literature search using the PubMed database. The search keywords were CAIX, G250 and/or renal carcinoma. We restricted our research to English language publications. We completed our investigations with specific additional keywords such as CAIX and pembrozilumab, ipilimumab, nivolumab, avelumab.

## 3. Evidence Synthesis

### 3.1. Role of CAIX in RCC Pathophysiology

CAIX is a trans-membranous protein that is expressed under physiological conditions in certain tissues, essentially the digestive track. Its presence in epithelial cells of the small bowel, duodenum, gastric mucosa and biliary tree has been reported. Conversely, it is widely expressed in several tumors by the action of hypoxia, described in many different types of cancers, such as lung, colorectal, gastric, pancreatic, breast, cervix, bladder, ovaries, brain, head and neck, astrocytomas, and oral cavity cancers [4,5].

Like other trans-membranous proteins, it is made up of three parts: an extracellular proteoglycan-like domain with the carbonate anhydrase (CA), a transmembrane segment and an intracytoplasmic part [6]. CAIX is involved in pH regulation of the cell environment [7].

The external side is responsible for the extracellular reversible catalyzation of the CO_2_ into bicarbonate by hydration. Transmembrane reabsorption of HCO_3_^–^ leading to an accumulation of protons in extracellular sector. The acidic cell microenvironment would favorize neo-angiogenesis by stimulating the vascular endothelial growth factor (VEGF) pathway. The internal side contains potential phosphorylation sites, permitting intracellular transduction signals. CAIX has a role on glucose metabolism through its cytoplasmic tail when phosphorylated.

The CAIX expression is regulated by a hypoxia-inducible factor 1 alpha (HIF1-α) [8]: under physiological normoxic conditions, HIF1-α is bound by the Von Hippel Lindau protein (pVHL) leading to its destruction via the ubiquitination and proteasome pathway) [9].

Under hypoxic stress, the affinity between HIF1-α and pVHL is decreased leading to an accumulation of cytoplasmic HIF1-α. This latter will be able to bind HIF1-β to form the HIF1 complex. HIF1 activates the transcription of several hypoxia-inducible genes that contain hypoxia responsive elements (HRE), like those involved in iron metabolism, glucose metabolism, angiogenesis (VEGF) and pH regulation (among which CAIX gene).

In the precise case of ccRCC (clear cell renal cell carcinoma), up to 95% of the tumor genomes are constitutionally VHL gene muted or deleted, thus leading to constitutive activation of HIF1 and CAIX upregulation, even in normoxic conditions.

The comparison of cDNA sequences between cancer and normal cells does not reveal any mutations or alternative splicing [10], and therefore the pathological mutations appear upstream in the signaling pathway. CAIX is also involved in cell adhesion thanks to its capacity to bind β-cathenin leading to a diminishing aptitude to capture E-cadherin which could eventually facilitate the cell migration and spreading [11].

### 3.2. CAIX as a Diagnostic Marker

#### 3.2.1. In Immunohistochemistry (IHC)

##### CAIX IHC Detection Permits Diagnosis of Histological Subtype

Renal cell carcinoma (RCC) covers many different subtypes, following the example of the recent new classification (2016 World Health Organization classification) [3]. Also, the pathological diagnosis of RCC subtype relies on the utilization of several tools, among which immunohistochemistry appears nowadays to be essential.

**Figure 1:**
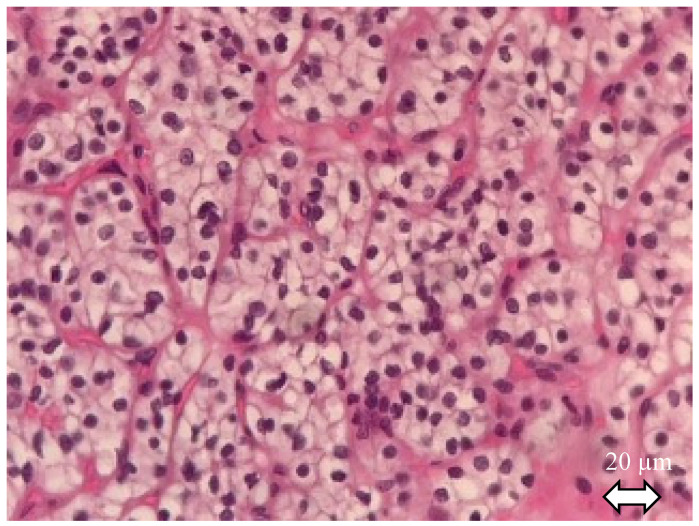
Clear cell renal cell carcinoma. Hematoxylin and eosin staining. The tumor shows solid alveolar growth pattern with regular network of small thin-walled blood vessels. Neoplastic cells have clear cytoplasm filled with lipids and surrounded by distinct cell membranes.

Making a precise diagnosis of the subtype is crucial given the wide alterity of related prognosis. After gross description, diagnosis of RCC, with histological features of the tissue sample, is always the first step before subtype identifying [12]: RCC tumor cells have abundant cytoplasm that is vacuolated, fluffy or granular, usually with indistinct cell borders (chromophobe renal cell carcinoma has distinct borders). Tumor nuclei have variable atypia, irregular contours, haphazard orientation with abnormal chromatin and variably prominent nucleoli (Figure 1).

The second step lies in immunohistochemistry analyses, principally based on three immunomarkers: cytokeratin 7 (CK7), α-methylacyl-CoA racemase (AMACR) and CAIX. These three markers could constitute an initial panel for diagnosis. Kim et al. [13] proposed a two-step strategy for the use of immunostaining in renal cell carcinoma subtype diagnosis: first with the triple panel described above (CK7, AMACR and CAIX) and then, if necessary, with others markers like c-kit, CD10 or cathepsin-K. Another team proposed to enlarge the panel adding the detection of TFE3 (transcription factor binding to IGHM enhancer 3) in IHC, permitting to detect the Xp11 translocation associated RCC [14,15].

The subtypes that stain with CAIX are mainly the clear cell renal cell carcinoma (ccRCC), which is the most common subtype, about 70% of RCC [16], the clear-cell papillary renal cell carcinoma (ccpRCC), and sometimes, with often moderate intensity, the non-type 1 papillary renal cell carcinoma (pRCCII).

**Figure 2:**
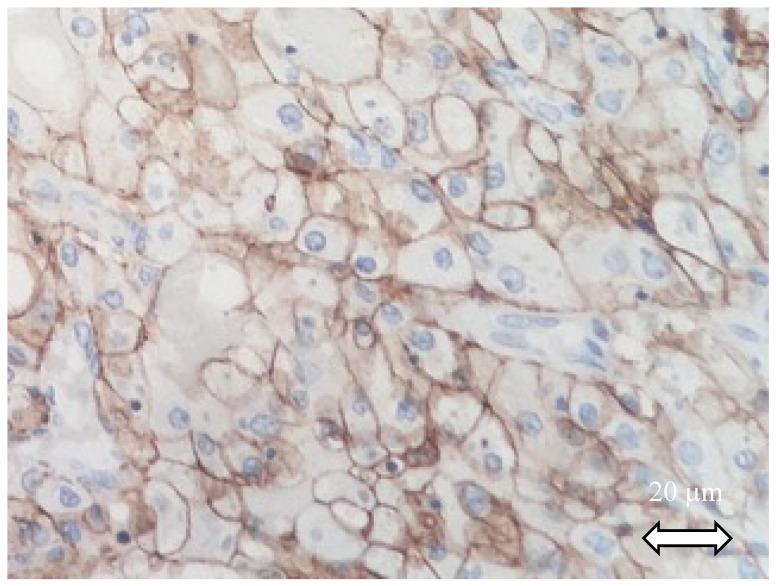
CAIX staining of a clear cell renal cell carcinoma. Tumor cells exhibit diffuse CAIX membranous immunoreactivity.

It is described that in almost all RCC subtypes, CAIX staining inversely correlates with CK7: the type 1 papillary renal cell carcinoma (pRCCI) is always CK7 positive and CAIX negative. Except for the renal neoplasm of the low-malignant-potential group (ccpRCC and multilocular cystic renal neoplasm of low malignant potential), where both may be positive. ccRCC is considered CAIX positive in IHC. It has also been observed that CAIX is diffusely and intensively staining with a box pattern in ccRCC in 75 to 100% of cases (described as “surrounding each quadrangle border”) when the CAIX may be focally stained in 25% of cases (Figure 2).

**Figure 3:**
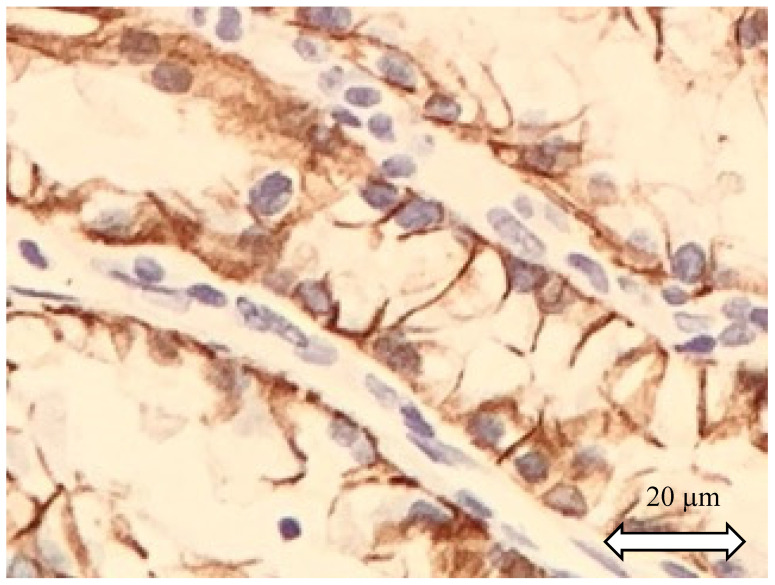
Clear cell papillary renal cell carcinoma. Cells display CA IX positivity with cup-like distribution.

Conversely, ccpRCC, where CAIX is often positive, manifests a specific cup-shaped staining of CAIX, with open lumen (Figure 3). ccRCC seems to have the highest expression of CAIX, probably due to the constitutional loss of VHL. As described above, ccRCC could be considered as always CAIX positive [17], while chRCC and pRCC1 are typically CAIX negative [18].

##### Prognostic Implication of CAIX Detection in IHC

As seen above, CAIX detection plays a major role in histological subtype detection. As the prognostic and evolution of the disease is narrowly correlated with RCC subtype and histological features, CAIX detection in IHC could give crucial prognostic information. Moreover, the principal rational is that High CAIX expression could witness the VHL-associated tumorigenesis, and ccRCC presenting an abnormally low CAIX rate could reflect the tumor de-differentiation and aggressiveness.

Ingels et al. realized a retrospective study on 143 extirpated local ccRCC, and tested 6 markers in IHC on the tumor’s samples: CAIX, c-MYC, Ki67, p53, vimentin and PTEN. The results showed that low CAIX expression (under 30%) and vimentin over-expression (over 50%) were associated with the worst outcomes (overall survival, progression-free survival and disease-free survival). The association of the two biomarkers in IHC (CAIX-/vimentin+) showed a good concordance [19].

Bui et al. [20] described unfavorable disease course in ccRCC with low CAIX expression levels in a study with 321 patients. Zhang et al. [21] and Zerati et al. [22] could not confirm this observation in 730 patients with ccRCC. They suggested that CAIX was not an independent prognostic marker for ccRCC after adjusting for nuclear grade or coagulative tumor necrosis.

Buschek et al. [23] retrospectively analyzed 1809 RCC according to the 2016 WHO classification criteria. CAIX Immunostaining were used in tissue microarray format. Four spots were analyzed for each tumor, so it could not perfectly represent the heterogeneity of the tumors. The staining signal was considered either negative, weak, moderate or strong. For the cohort of ccRCC (1101 tumors), only the ones with strong staining were considered positive. Any pRCC tumor with weak, moderate or strong staining was considered positive (any kind of positive staining). High CAIX expression in the ccRCC group was associated with low ISUP and Furhman grade, low tumor stage and absence of distant metastases. It was also associated with better recurrence-free survival and overall survival. In the pRCC group (252 tumors), only trends were observed between CAIX expression and the clinical-pathological tumor parameters. High expression of CAIX was associated with diminished recurrence-free survival. In other terms, high CAIX expression in ccRCC seemed to be associated with better prognosis, while high CAIX expression in pRCC seemed to be unfavorable.

One meta-analysis [24] treated CAIX expression and clinico-pathological issues. Four studies were mainly retained, namely the articles from Bui et al., Zhang et al. (mentioned above), Patard et al. [25] and Chamie et al. [26] High CAIX expression seemed to be associated with lower tumor stage (pT1T2 vs. pT3T4), lower tumor grade (G1G2 vs. G3G4), absence of nodal involvement (N0 vs. N+) and favorable ECOG score (0 vs. ≥1).

In 2003, Bui et al. demonstrated that 85% of tumor surface staining represents a good cut-off threshold to discriminate high vs. weak immunohistochemistry CAIX staining and to predict survival [20]. In 2014, Ingels et al. found 30% was the best threshold [19].

Chamie et al. proposed another score to measure the CAIX expression: it associates the percentage of stained surface with the staining intensity. This scores range from 0 to 300 (intensity 0 to 3, semiquantitative assessment). A high CAIX score (>200) was associated with prolonged DFS and OS [26]. Evidence on CAIX expression measured by immunohistochemistry and clinical outcomes is summarized in Table 1.

We could hypothesize that an “aberrant” expression of CAIX in RCC tumors exists, which seems to be associated with poor prognosis, may reflect cancer dedifferentiation.

##### CAIX Measured by Immunohistochemistry and Systemic Treatment Response Prediction

Tostain et al. [27] and Atkins et al. [28] analyzed the correlation between CAIX high expression in RCC metastatic patients and increased tumor sensitivity to interleukin-2 (IL-2). This treatment is no longer recommended in metastatic RCC management [29].

Choueiri et al. [30] assessed the utility of CAIX expression to predict the response in metastatic RCC patients treated with sunitinib or sorafenib, but lacked to enlighten a prognostic value. However, they showed that patients presenting low CAIX expression may benefit less from sorafenib. In another study from the same authors [31], no correlations between CAIX expression and response to sorafenib or IL-2 were observed, leading to the conclusion that CAIX status had no prognostic value. To our knowledge, no studies have been performed so far to assess new immunotherapies treatment such as nivolumab, ipilimumab, pembrozilumab or avelumab through CAIX expression.

#### 3.2.2. Value of Circulating CAIX

##### Diagnostic Value

Lucarini et al. [32] evaluated the potential of circulating CAIX to diagnose ccRCC. The concentration of circulating CAIX was significantly higher in patients presenting ccRCC in comparison to controls (benign tumors or healthy subjects). The CAIX enzymatic activity showed no differences between the three groups (eight patients each). Pinrinççi et al. [33] also showed significantly increased levels of circulating CAIX in patients afflicted by ccRCC, compared to controls.

Blood sampling of patients with indeterminate tumors on imaging could give an indication of the nature of this tumor.

##### Prognostic Value

Li et al. measured the plasma levels of CAIX at diagnosis (for histologically proven RCC). Comparing 91 RCC proven patients to 32 controls, they showed a significantly increased level of CAIX in RCC patients compared to controls [34]. Moreover, metastatic patients had higher rates of serum-circulating CAIX compared to those who had localized diseases. The median follow-up was 38 months for patients with a localized, surgically-managed disease. The comparison in plasmatic levels of CAIX showed higher rates in patients who experienced recurrence during follow-up, suggesting that an increased level of plasmatic CAIX at diagnosis is predictive of recurrence.

From another perspective, Liu et al. [35] developed an alternative technique to detect circulating tumor cells using ligands of membranous CAIX designed for RCC patients. They showed a significant positive association between circulating CAIX-RCC cells and disease progression, histological features and clinical staging. These results demonstrate the correlation between the serum levels of CAIX, RCC circulating cells and the tumor burden, aggressiveness and propensity of recurrence.

#### 3.2.3. CAIX and Imaging

Current developments in molecular imaging taking advantage of biomarker tracking can overcome the limitations of conventional imaging (CT and MRI) for renal masses exploration. Those are often serendipitous and rarely permit a reliable prediction of tumor subtype or malignancy. Tumor biopsies have their technical limits: they are not contributive in more than 20% of cases [36]. By focusing on a very limited area they do not reflect the tumor heterogeneity [37]. The contribution of ^18^F-FDG PET/CT has been well-described [38]. Although it might detect RCC metastasis, it does not work for primary renal tumors and is not recommended in RCC exploration [39]. A recent molecular imaging tool targeting CAIX is girentuximab [40], also called cG250, an anti-CAIX chimeric antibody which allows PET/CT imaging when bonded with certain isotopes (^131^I, ^124^I or ^111^In) that furthermore leads a pathway to radioimmunotherapy (RIT) in ccRCC.

Another diagnostic approach employs ultrasound molecular imaging (USMI), using CAIX-targeted bubbles, allowing the detection of masses that express the marker. Facility, accessibility and innocuity are solid arguments for this technique [41]. Table 2 summarizes the evidence on the clinical value of CAIX targeting in molecular imaging.

##### CAIX and Conventional CT Scans

Young et al. published a study aiming to assess the correlation between the gross appearance of tumoral blood vessels on injected CT scans with the CAIX expression intensity in IHC [42]. The CAIX score (a technique developed by Chamie et al., described above) was determined by immunostaining on 105 ccRCC tumors.

High expression (range 200–300) of CAIX was more likely to show gross appearance of vessels on CT scan. The 4 phases CT-scan could potentially predict CAIX expression, correlated to the neovascularization propensity. Nonetheless, the other CT features did not correlate with the CAIX score (i.e., pattern of enhancement, necrosis, renal vein invasion).

##### Positron Emission Tomography

Lindenberg et al. [43] evaluated the potential of girentuximab, labeled with ^124^I when used as a PET/CT agent. They tested the radiotracer in a study with 195 patients scheduled for renal mass resection. The analyses showed 86% sensitivity and 86% specificity for detection of malignant tumor of the kidney (respectively 76% and 47% for contrast enhanced CT).

Hekman et al. [44] reported a clinical study using girentuximab labeled with ^89^Zr in PET/CT imaging. They showed a good capacity of ^89^Zr-girentuximab to predict ccRCC histology subtype in recently diagnosed renal masses, with all resected ccRCC being pre-operatively positive on molecular imaging. It also presented a good performance to rule out recurrence for previously treated patients, with no lesion progression or metastasis detected with enhanced CT-scan when ^89^Zr-gurentuximab PET-CT was negative.

Minn et al. [45] developed a novel dual motif ligand for PET/CT detection of localized and metastatic ccRCC. ^64^Cu-XYIMSR-06 is a small size synthesized molecule added with radioactive copper, presenting high affinity for CAIX. Experiments in vitro and in nude mice bearing human ccRCC xenografts showed interesting results, notably the advantageous pharmacokinetics facilitating the PET/CT imaging schedules compared to girentuximab: PET/CT acquisition 1 h to 1 day after injection vs. 2 to 6 days, respectively. No human utilization has been described yet.

Turkbey et al. [46] reported the results of a phase 2 clinical trial on a small molecule radiotracer targeting CAIX: ^18^F-VM4-037. Due to physiologic renal excretion, the tracer showed a low capacity to detect primary renal lesions (mean SUV 2.55; range 0.58–4.23, SD: 1.23). On the other hand, the detection of ccRCC metastasis was remarkable (mean SUV 5.92), comparable to 18F-FDG PET/CT imaging for detection of extra renal lesions.

##### Ultrasound Molecular Imaging (USMI)

Zhu et al. described a new USMI contrast product: CAIX aptamer-functionalized targeted nanobubbles [41]. As seen above, CAIX may be expressed by many different malignancies and it is a trans-membranous protein. The nanobubbles present a size specificity permitting the passive absorption by the tissues realizing an intratumoral cell targeting, where conventional microbubbles stay blocked in the vessels. They created the CAIX aptamer using a repeated PCR technique (a selection of one single-stranded DNA by screening the systematic evolution of ligands by exponential enrichment, 10 rounds). They tested the CAIX nanobubble USMI on xenografted nude mice with different malignant cell-lines. Human renal cell carcinoma 786-O cells and HeLa human cervical cancer cells, which are known to be CAIX-positive, and BPxPC-3 human pancreatic cancer cells, which are not for control. They also described the distribution, loading and binding ability of these nanobubbles, demonstrated by immunofluorescence. They controlled their experimentation with nanobubbles attached to a nonsense aptamer. The experimentation showed the ability of the nanobubbles to reach the tumor parenchyma, in addition to the tumor vascularization enhancing. The tumor CAIX immunostaining matched with the targeted nano-bubbles localization.

##### Fluorescence for Facilitation of Peroperative Recognition

CAIX-targeted agents are developed in order to facilitate the per operative recognition of small renal masses. This could allow for safer surgery and better resection margins when partial nephrectomy is indicated, with an objective to diminish the recurrence rate.

Muselaers et al. [47] first realized a preclinical study to validate the concept of guided surgery with G250, a dual labeling agent with ^111^In and a near infrared fluorophore (IRDye800CW). BALB/c nu/nu mice with intraperitoneal human derived ccRCC cellular lines received one injection of the CAIX targeting agent or a dual labeled irrelevant control antibody (four mice per group), and subsequently underwent a PET/CT scan once a week during five weeks. Fluorescence imaging were determined with ex vivo biodistribution studies. Results showed good concordance between PET/CT imaging, with maximum uptake of the radiotracer in the first week and fluorescence, with excellent delineation of the tumors.

Hekman et al. [48] subsequently tested the technique in a first-in-human phase 1 study, published in 2018. Dose escalation (5, 10, 30, or 50 mg) of CAIX-targeting antibody ^111^In-DOTA-girentuximab-IRDye800CW was performed in a cohort of 15 patients scheduled for small renal mass surgery. PET/CT was performed four days after injection and surgical intervention at day 7 with a gamma probe and a near-infrared fluorescence camera. Histological outcomes showed 12 ccRCC, all hyperfluorescent at any dose, all PET/CT positives and all presenting a stronger signal with per operative gamma camera. No study-related serious adverse events were noticed.

### 3.3. CAIX in Therapeutics

Several RCC therapeutic strategies focus on CAIX. It is a highly-expressed, specific surface antigen of ccRCC, allowing targeted treatments: vaccines and immunity sensitization or radioimmunotherapy using cG250/girentuximab. Blocking the CAIX expression in ccRCC might sensitize the tumor to external radiotherapy, reducing its pH regulation skills.

#### 3.3.1. Vaccines and Immune-Mediating

The cell-mediated activation of anti-tumoral immunity remains in three steps: antigen presentation, activation of co-stimulatory signals and amplification of the immune response. Current research on these therapeutics remain mainly preclinical, awaiting in-human studies.

In 2008 Shuch et al. [49] reported a new vaccine strategies. The main one was the use of dendritic cells (DCs, specific tumor antigen presentation) via an introduction of a granulocyte-macrophage colony stimulating factor (GMCSF)-CAIX fusion protein (produced by viral bioengineering), in order to activate DCs and thus trigger a CD8+ mediated, CAIX targeted, antitumoral response. Kim et al. [50] presented the results of a pre-clinical study in 2011 aiming to activate DCs by a combination of CAIX and *Acinetobacter baumannii* outer membrane protein A (AbOmpA) in a murine model [51]. A significant immunostimulatory of DCs was observed via secretion of IL-2 and interferon (IFN)γ in T-cells. In 2013, Birkhäuser et al. [52] tested a dendritic cell vaccine in immunocompetent mice, showing encouraging results with significative tumoral growth inhibition, specifically in CAIX positives tumors.

In 2018, a phase 1, open-label, dose-escalation and cohort expansion study evaluated the safety and immune response to autologous dendritic cells transduced with AdGMCA9 (recombinant adenovirus encoding the GMCSF-CAIX fusion gene) in patients with metastatic renal cell carcinoma [53]. 15 patients were enrolled, among which nine received the planned treatment. They did not present any serious adverse event. This phase 1 protocol did not permit any efficiency statement.

Chang et al. [54] showed in a preclinical study the ability of human anti-CAIX antibodies to mediate immune cell inhibition of renal cell carcinoma. They demonstrated that human anti-CAIX mAbs fixation on CAIX expressive RCC led to an immune-mediated destruction of tumoral cells in vitro by antibody-dependent cell-mediated cytotoxicity (ADCC), complement-dependent cytotoxicity (CDC) and antibody-dependent cellular phagocytosis (ADCP). They also showed a migration inhibition of RCC cells in vitro. Administration of the same anti-CAIX human mAbs in an orthotopic RCC model utilizing allogeneic human peripheral blood mononuclear cells in NOD/SCID/ IL2Rγ−/− mice showed inhibition of tumor growth.

#### 3.3.2. cG250/Girentuximab and Radioimmunotherapy

Oosterwijk et al. [55] published in 2011 the results of a preclinical study on nude mice bearing human RCC xenograft. The objective was to observe the effect of several tyrosine kinase inhibitors (TKIs): Sunitib, sorafenib or vandetanib on the bio-distribution of injected marked ^125^I-gerentuximab. Tumor growth and vascularization were significatively affected, probably due to the TKI therapy, however ^125^I-girentuximab accumulation in the tumor were drastically diminished in vivo and at gamma-detection. Nonetheless, the ^125^I-gerentuximab tumor-accumulation recovered after several days of TKI discontinuation. We should consider major interactions between cG250 and TKIs that must impose precaution in further trials testing cG250 on humans being treated. In 2013, the same team reviewed the state of the art concerning radioimmunotherapy using cG250/girentuximab labeled with radioisotopes in RCC as promising treatment [56]. Clinical studies realized between 1998 and 2011 were screened: seven phase I, three phase II (in metastatic RCC) and 1 phase III (in adjuvant setting for patients at high risk after nephrectomy, the ARISER study); showing limited benefice and suggesting a better efficiency for small-volume patients.

After Stillbroer et al. [57] determined the maximum tolerated dose of 177Lu-girentuximab in a phase I study, Muselaers et al. [58] evaluated in 2015, in a phase II non-randomized single-arm trial, the efficacy of ^177^Lu-girentuximab. Fourteen metastatic ccRCC patients with evidence of progressive disease were enrolled between April 2011 and August 2014. They received an ^177^Lu-girentuximab infusion (2405 MBq/m^2^), then clinical and radiological outcomes, according to the Response Evaluation Criteria in Solid Tumors (RECIST v1.1), were prospectively assessed. At first evaluation after the first infusion, eight patients (57%) had stable disease (SD) and 1 (7%) had partial response (PR). Hematological issues (prolonged low blood cell count) were the major adverse event (grade 3 or 4 myelotoxicity observed in almost all patients): five patients on six receiving the second infusion (75% of initial dose) had SD but prolonged thrombocytopenia, imposing treatment discontinuation.

The combined myelosuppressive activity of both TKIs and girentuximab might be a major obstacle for further development of this strategy [59].

#### 3.3.3. Sensitization to Radiotherapy Inhibiting CAIX Expression

Duivenvoorden et al. [60] published a preclinical study suggesting the potential protective role of CAIX for irradiated tumors, as CAIX is a pH regulator. Introduction of a pharmacological CAIX inhibitor, or transfection with shRNA-mediated knockdown of CAIX, in xenografted nude mice with ccRCC (786-O cells) resulted in a better response (in vitro) to irradiation (6Gy), compared with mice receiving either irradiation or pharmacological alone. The tumors were significantly smaller in transfected mice (in vivo).

## 4. Conclusions

In conclusion, the place of CAIX remain prevalent from diagnosis to treatment and treatment response monitoring, especially for the clear cell subtype, the most common form of RCC. While the value of CAIX in immunohistochemistry is well established, the development of molecular imaging or treatment applications have not yet passed phase III clinical trial validations and remain more or less exploratory. However, rationale and first studies outcomes are encouraging and open the way for new advances in the management of the disease.

## Figures and Tables

**Table 1:** Evidence synthesis of CAIX application for immunohistochemistry and its predictive value for clinical outcomes.

Common CAIX Expression in Immunohistochemistry		
RCC subtype	CAIX	CK7
ccRCC	+++	−
pRCCI	−	+
pRCCII	+/−	+/−
chRCC	−	+
ccpRCC	+	+
multilocular cystic renal neoplasms	+/−	+
Predictive value of CAIX expression		
Study	RCC subtype	Results
Ingels et al. [19]	ccRCC	CAIX expression < 30% in IHC associated with diminished OS, PFS and DFS
Bui et al. [20]	ccRCC	Low CAIX expression associated with unfavorable disease course
Buschek et al. [23]	ccRCC	High CAIX expression was associated with better outcomes
Buschek et al. [23]	pRCC	Abnormal expression of CAIX was associated with diminished PFS
Samberkar et al. [24]	ccRCC	High CAIX expression associated with lower tumor grade and stage, N0, favorable ECOG score
Chamie et al. [26]	ccRCC	High CAIX score > 200 associated with prolonged DFS and OS

ccRCC: clear cell renal cell carcinoma. pRCCI: papillary renal cell carcinoma. pRCCII: papillary renal cell carcinoma. chRCC: chromophobe renal cell caricnoma. ccpRCC: clear cell papillary renal cell carcinoma. OS: overall survival. PFS: progression free survival. DFS: disease free survival. ECOG: Eastern cooperative oncology group. CAIX: carbonic ahydrase IX. CK7: cytokeratin 7. +++: expression is almost systematic +: classically positive −: classically negative +/−: variable.

**Table 2:** Evidence synthesis of CAIX application for molecular imaging.

USMI			
Study	product	abilities	limitations
Zhu et al. [41]	CAIX aptamer functionalized nanobubbles	Enhance vascularization and parenchyma in CAIX-expressive tumors	Preclinical data only
PET imaging			
Study	product	abilities	limitations
Lindenberg et al. [43] Hekman et al. [44]	Girentuximab/cG250	Good sensitivity and specificity for ccRCC many labeling possibilities with several isotopes (124I, 131I, 111In, 89Zr etc.)	Slow pharmacokinetics, acquisition within 2 to 6 days
Lindenberg et al. [43]	18FDG	Detection of metastasis	Low sensitivity for primary tumor
Minn et al. [45]	64Cu-XYIMSR-06	Fast pharmacokinetics, acquisition within 1 h to 1 day	Preclinical data only
Turkbey et al. [46]	18F-VM4-037	Sensitivity for metastasis detection could be better than 18FDG	Low sensitivity for primary tumor

USMI: ultrasound molecular imaging. PET: positron emission tomography. Girentuximab: chimeric IgG1 monoclonal antibody to carbonic anhydrase IX. 18FDG: 18-flurodesoxyglucose. 18F-VM4-037 is a small molecule radiotracer targeting carbonic anhydrase IX. 64Cu-XYIMSR-06 is a small molecule radiotracer targeting carbonic anhydrase IX.

## Abbreviations

AbOmpA	*Acinetobacter baumannii* outer membrane protein A
ADCC	Antibody-dependent cell-mediated cytotoxicity
AdGMCA9	adenovirus encoding the GMCSF-CAIX fusion gene
ADCP	Antibody-dependent cellular phagocytosis
AMACR	α-Methylacyl-CoA racemase
CAIX	Carbonic anhydrase IX
ccpRCC	Multilocular cystic renal neoplasm of low malignant potential
ccRCC	Clear cell renal cell carcinoma
CDC	Complement-dependent cytotoxicity
chRCC	Chromophobe renal cell carcinoma
CK	Cytokeratine
CT	Computed tomography
DC	Dendritic cell
FDG	Fluorodesoxyglucose
GMCF	Granulocyte-macrophage colony stimulating factor
HIF	Hypoxia inducible factor
HRE	Hypoxia responsive elements
IHC	Immunohistochemistry
MRI	Magnetic resonance imaging
PET	Positron emission tomography
pRCC	Papillary renal cell carcinoma
RCC	Renal cell carcinoma
pVHL	Von Hippel Lindau protein
RECIST	Response evaluation criteria in solid tumors
SD	Standard deviation
SUV	Standardized Uptake Value
TKI	Tyrosine kinase inhibitor
TFE3	Transcription factor binding to IGHM enhancer 3
USMI	Ultrasound molecular imaging
VEGFR	Vascular endothelial growth factor
WHO	World Health Organization
